# Active surveillance for KPC-experiencereport

**DOI:** 10.1186/1753-6561-5-S6-P141

**Published:** 2011-06-29

**Authors:** CT Zogheib, MM Baraldi, MM Simonetti, CM Santoro

**Affiliations:** 1 Serviço de Controle de Infecção Hospitalar, Hosptial Alemão Oswaldo Cruz, São Paulo, Brazil

## Introduction / objectives

Bacterial resistance to antimicrobials has increased over the years, but the importance of empirical investigation of these bacteria contributes to the prevention of spread within the institution. The Infection Control Comission Protocol has as surveillance for resistant organisms since 1996.In Brazil, Klebsiela pneumoniae carbapenemase enzyme (KPC) was the first described in 2005 with an increased number of cases in the last year, prompting us to seek the epidemiological profile of inpatients.

## Methods

Observational study in a general hospital for investigation of asymptomatic KPC, in two periods with an interval of three months between October 2010 and February 2011, with population as inpatients in the Intensive Care Unit and Medical Clinics with time hospitalization exceeding 20 days or Precaution Contact by resistant microorganisms, which were held in four anal swab samples.

## Results

We selected a total of 108 patients, 20 of these (18%) were excluded from the study, 13 (12%) were discharged from hospital, 5 (5%) died, 2 (2%) were transferred to another hospital. A total of 128 samples were included, where seven (5.4%) tested positive for KPC and 4 (4.5%) patients were colonized.

## Conclusion

Actions surveillance to identify resistant organisms, implementation of precaution-based transmission mode, adopting the policy of hand hygiene using alcohol gel at the point of care, allow for the prevention of spread among patients with risk factors such as prolonged hospitalization, immunosuppression and the use of many antibiotics.

## Disclosure of interest

None declared.

